# Severe Primary HSV-2 in a Perinatal HIV-Infected Woman with Advanced Immunosuppression

**DOI:** 10.1155/2012/346039

**Published:** 2012-08-01

**Authors:** Lana Lee, Allison Agwu, Nancy Hutton

**Affiliations:** ^1^Division of General Pediatrics and Adolescent Medicine, Department of Pediatrics, Johns Hopkins School of Medicine, Baltimore, MD 21287, USA; ^2^Divisions of Adult and Pediatric Infectious Diseases, Departments of Internal Medicine and Pediatrics, Johns Hopkins School of Medicine, Baltimore, MD 21287, USA

## Abstract

Herpes simplex virus type 2 (HSV-2) is most commonly associated with mucocutaneous manifestations; however, coinfections with HIV may be associated with atypical and more severe presentations of clinical disease. We present a case of a young woman with advanced perinatally acquired AIDS presenting with severe purulent pharyngitis, fevers, and toxic appearance with a subsequent diagnosis of disseminated primary HSV-2 infection in multiple noncontiguous mucocutaneous sites. This case highlights an unusual presentation of the protean nature of primary HSV infection and the potential severity of illness in patients with advanced immunosuppression.

## 1. Introduction

Herpes simplex virus type 2 (HSV-2) is one of the most prevalent sexually transmitted infections worldwide with seroprevalence rates ranging from 30% up to 90% in patients with HIV [1–3]. Although most commonly associated with recurrent mucocutaneous outbreaks, atypical presentations of HSV-2 in individuals coinfected with HIV can be associated with more severe disseminated clinical presentations such as meningoencephalitis, hepatitis, and pneumonitis [4]. We present a case of a young woman with advanced AIDS who presents with severe concurrent primary HSV-2 pharyngeal and genital ulcerations necessitating hospitalization.

## 2. Case Report

A 24-year-old African-American female with perinatally acquired HIV and AIDS (CD4 = 27, VL 16, 164) with nonadherence to highly active antiretroviral therapy (HAART), presented to her primary HIV provider with sore throat, fever, and weakness for two weeks, and URI symptoms for one week. She reported difficulty with swallowing solids and liquids including all HAART pills due to throat pain. Three days prior to presentation, she was seen at an outside emergency department and had a positive rapid streptococcal antigen test for which she was prescribed amoxicillinclavulanic acid for strep pharyngitis; she did not fill the prescription. On exam, there was a 6-kilogram weight loss since two months prior to presentation. She was febrile and was noted to have a muffled voice. There were thick white plaques on the left tonsil, tonsillar pillar, and tongue mucosa that did not scrape off. Due to the degree of discomfort and persistent fevers up to 102 degrees Fahrenheit, there was concern for a severe infection of the parapharyngeal or retropharyngeal spaces (i.e., Lemierre's syndrome, abscess), and a CT scan of the neck was obtained. It revealed bilateral palatine tonsillitis with evidence of a possible early abscess in the left glossopalatine recess, but no obvious areas of fluid collections. Clindamycin was added to the antibiotic regimen at that time.

Her past medical history was significant for a CD4 nadir of 14 cells/mm^3^, recurrent skin infections and abscesses, chronic purulent otitis media requiring tympanostomy tubes, trichomonas, and Grade 1 cervical intraepithelial neoplasia. She was notably nonadherent to her HAART regimen of daily atazanavir, lamivudine, abacavir, and ritonavir. She was also on monthly aerosolized pentamidine for pneumocystis prophylaxis, but was not taking prophylaxis for mycobacterium avium intracellulare.

Three days later, the throat pain had worsened with extension to the right side. The patient remained febrile and was unable to take any solids or liquids orally due to pain. She also reported the development of a painful genital ulcer during the past week. She had vaginal intercourse with an asymptomatic male partner one week prior to the onset of symptoms and reported intermittent condom use. She denied any new sexual partners for the last 2 months and adamantly denied orogenital sexual contact.

The patient denied headaches, visual changes, vomiting, seizures, or other focal neurologic symptoms on review of systems.

Physical exam at this time revealed an ill-appearing young woman who was alert, and oriented with new enlargement of the right tonsil with erythema, new white plaques on the posterior pharynx without improvement of the left tonsil or the left side of the tongue. The patient demonstrated pain when opening her mouth for exam. On the genitourinary exam, there was labial swelling and a 1 cm large open ulcer with cream-colored discharge centrally over the ulcer. There was also a creamy film covering the vaginal introitus. She was unable to tolerate a speculum and one-finger manual exam due to significant swelling and pain.

The patient was admitted to the hospital for worsening tonsillitis and pharyngitis not responding to dual oral antibiotic coverage, dehydration, and persistent fevers. She was started on intravenous ampicillin sulbactam, fluconazole, and acyclovir. Subsequent viral cultures from the throat, mouth, and genital ulcer became positive for HSV-2 at two days. Two years prior to the current presentation, qualitative determination of antibody status for specific IgG to both HSV-1 and HSV-2 using indirect chemiluminescence immunoassays was negative during the patient's pregnancy. Repeated evaluation using the same method for specific IgG to both HSV-1 and HSV-2 remained negative with the current illness at approximately two and a half weeks from onset of symptoms. Antibody status of HSV IgM was also negative. She was known to have had appropriate antibody responses to Hepatitis A and B vaccines. Other admission labs included a white blood cell count of 8600 per cubic millimeter with an absolute neutrophil count of 7040 per cubic millimeter, and normal complete metabolic panel with normal liver function markers.

The patient's condition improved with treatment and once she was able to tolerate oral intake, she was discharged on hospital day 5 on oral valacyclovir 1000 mg twice daily, fluconazole 200 mg daily, and amoxicillin clavulanic acid extended-release 1 gram twice daily.

## 3. Discussion

The herpes simplex viruses are large double-stranded DNA viruses belonging to the Herpes viridae family. Transmission typically occurs through close personal contact from infected individuals through mucosal surfaces or abraded skin. HSV-1 is generally associated with oropharyngeal mucosal infections and is frequently acquired during childhood, while HSV-2 is the most common cause of genital herpes and typically acquired through sexual contact. However, studies have demonstrated that there is a significant overlap in the epidemiology and clinical manifestations of HSV-1 and HSV-2 [5, 6].

Clinical presentations with a primary infection of genital herpes can range from asymptomatic to formation of painful lesions with or without other associated symptoms. Women, in particular, present with a higher frequency of systemic symptoms compared to men (68% versus 39%), which may be characterized by headache, fever, malaise, and myalgias [7]. While localized symptoms may involve pain or itching, dysuria, vaginal or urethral discharge, and tender inguinal adenopathy, complications can extend to include aseptic meningitis, paresthesias of the leg and perineum, other mucocutaneous lesions beyond the genital area, pharyngitis, esophagitis and visceral dissemination [4, 7].

HSV pharyngitis is not uncommon among patients presenting with primary genital herpes, although, interestingly, our patient developed pharyngitis symptoms prior to presenting with genital lesions [4, 7]. The several days of oral manifestations without genital lesions made the diagnosis of HSV more difficult. Individuals may complain of sore throat during the acute episode and clinical signs can range from mild erythema to a more severe diffuse ulcerative or exudative posterior pharyngitis, or tonsillitis. While a history of orogenital contact, which she adamantly denied, is a risk factor for development of pharyngeal infection, disseminated infection with the presence of pharyngitis in the absence of reported sexual contact has also been reported [8]. In one case series of patients with genital HSV infections, 11% of patients with a primary HSV-2 genital infection also had HSV-2 isolated from the pharynx. However, while almost half of all the patients with genital HSV infection in the case series reported performing recent oral sex (48% men, 51% women), the subset of patients with primary HSV-2 genital infections with concurrent pharyngeal infection who may have performed oral sex is not described [7]. Interestingly, in the same case series, several patients also had a prior recent diagnosis of streptococcal pharyngitis similar to our patient. Whether the mechanism is due to a bacterial superinfection of a herpetic ulcerative lesion or a synergistic relationship between HSV-2 and a bacterial infection causing a more severe clinical presentation it is not clear.

Many studies have demonstrated a synergistic relationship between HSV-2 and HIV acquisition and transmission [9]. There is also evidence demonstrating that the presence of coinfection with HSV-2 and HIV-1 is associated with atypical and more severe clinical presentations, as well as more frequent recurrences of secondary HSV reactivation and recurrence [10–12]. Additionally, there is also a relationship between greater degrees of immunosuppression and advanced HIV with more severe presentation of HSV disease [13].

Our patient did not demonstrate serologic evidence of prior HSV-2 infection or exposure. With the absence of IgG and IgM antibodies to both HSV-1 and HSV-2, and isolation of HSV-2 from viral cultures at both the pharyngeal and genital sites, this presentation is consistent with a primary HSV-2 infection simultaneously involving the genital area and pharynx. Given that our patient denied active lesions on her partner, we presume the patient acquired HSV-2 infection through asymptomatic shedding of the virus from her partner.

More needs to be known about the mechanisms of HSV-2 dissemination, particularly in the setting of severe immunosuppression and HIV/AIDS. The epidermal basement membrane has been shown to restrict the spread of HSV; however, this presentation of noncontiguous mucocutaneous spread in the absence of contact exposures or obvious autoinoculation is atypical [14]. Clinicians should remain vigilant in considering HSV in severely immunocompromised individuals even when bacterial infections are suspected as this may minimize delay in diagnosis, while impacting clinical management and subsequent outcomes. Although the inability to definitively exclude the possibility of any oro-genital contact is a limitation, this case raises an interesting question of the association of genital HSV-2 infection and extra-genital mucocutaneous spread.

## 4. Conclusion

Coinfections of HIV and HSV are common and may result in more severe and atypical presentations, particularly in individuals with severe immunocompromise. HSV-2 should therefore be considered early in the differential for infectious pathogens of oropharyngeal infections in individuals with HIV. Initiation of empiric treatment for HSV-2 while awaiting results from diagnostic testing should be considered. Understanding mechanisms of spread for dissemination of HSV, particularly in the setting of severely impaired immune responses seen in HIV/AIDS, would be an important consideration for management of primary HSV-2 genital infections.
